# Supplementary material for: Fluctuations in influenza-like illness epidemics and suicide mortality: A time-series regression of 13-year mortality data in South Korea
Source: PLoS One. 2021 Feb 12;16(2):e0244596. doi: 10.1371/journal.pone.0244596 (PMC7880447; doi:10.1371/journal.pone.0244596)
Supplement: S1 Box — (DOCX) [file pone.0244596.s001.docx]

S1 Box. Korean government’s guidelines for reporting “influenza-like illness”

| [Reporting range]: Influenza-like illness, including influenza patient  [Reporting period]: 7 days after the first diagnosis or visitation  [Diagnostic criteria]  For patient diagnosis: Clinical symptoms consistent with influenza and confirmed pathogen infection by one or more of the following criteria:   1. Virus isolation from specimen (throat, nasopharyngeal, throat, nasal aspiration) 2. Antibody titers of convalescent sera are more than 4 times higher than those of acute phase 3. Detection of influenza-specific antibodies in specimen 4. Detection of virus-specific genes in specimens (throat, nasopharyngeal, throat, nasal aspiration)   For influenza-like illness: The subject has a cough or sore throat with a fever of over 38°C |
| --- |
