# Supplementary material for: Fluctuations in influenza-like illness epidemics and suicide mortality: A time-series regression of 13-year mortality data in South Korea
Source: PLoS One. 2021 Feb 12;16(2):e0244596. doi: 10.1371/journal.pone.0244596 (PMC7880447; doi:10.1371/journal.pone.0244596)
Supplement: Attachment — Submitted filename: PONE-D-20-10691.pdf [file pone.0244596.s002.pdf]

Review on manuscript "Fluctuations in influenza epidemics and suicide mortality: A time-series regression of 13-year mortality data"

Summary of the manuscript

This manuscript investigates an association between suicide mortality and the rate of influenza-like illness (ILI) in Korea. The proposed study is exciting and timely as we experience a virus outbreak. Still, I am afraid that the presentation of statistical methods, data description, the measure of the unit of variables, and analysis is confusing.

I strongly recommend using consistent words for suicide mortality and ILI rate. Throughout the manuscript, I read suicide attempts, influenza symptoms, influenza infections were used instead of suicide mortality, and ILI rate that could distract any reader. Another confusing word was novel A(H1N1)pdm09. Do we have a difference between novel A(H1N1)pdm09, classic novel A(H1N1)pdm09, the new novel A(H1N1)pdm09, A(H1N1)pdm09? I think all are the same, but I would choose one name for the entire text.

Suggestions to the authors for major revision

1. Abstract:

   Objective: The title is entirely different from the objective of the study. 1) As I understood, this study is only in Korea, so I would recommend reflecting this in the title. 2) title says influenza epidemics and suicide mortality, but the objective is about influenza infections and neuropsychiatric symptoms.

   Aims: I would recommend being specific about the population under study - Korea.

   Methods: I couldn't understand the sentence that the regression coefficient for suicide based on the percentage change of influenza.

2. Lines 2-9: This study focused on the population of Korea, so why wouldn't the authors mention any previous study in Korea in the introduction?

3. Line 10: "In population studies ..." I would suggest being specific (name it) about the population.

4. Line 11: Do authors consider weekly/monthly suicide mortality? I would recommend saying the month instead of the spring season because seasons are different across countries.
5. Line 11: I would either use overlap or follow, not both.
6. Line 11-12: What are the traditional upper respiratory infections?
7. Line 14: I am not sure about using the "etiology of suicide." Could we use the cause of suicide mortality? Moreover, if the prevention strategies for Korea, then authors could revise the sentence to reflect this in the sentence.
8. Line 16: new 16 influenza (H1N1) virus pandemic and A(H1N1)pdm09 are different?
9. Line 16: "large" is a confusing adjective for the population.
10. Line 23: I would say subject information is blinded.
11. Line 24: What does "current study" refer to? Would it be a study in this paper? Correct me if I am wrong: I assume that this paper uses a retrospective study data.
12. Line 29-30: I would add ILI per 1000 visits weekly/monthly.

    Line 37: It is confusing that the introduction reads on neuropsychiatric symptoms, but this line picks only suicide. I would rewrite clearly whether this study considers only suicide mortality or the number of suicides per week or whatever appropriate throughout the text.
13. Line 40-41: Correct me if I am wrong. I assume that the authors didn't define the codes so that I would rewrite this sentence. For example, we chose cases with X60-X84 and Y870 related to intentional self-harm and sequelae of intentional self-harm, respectively.
14. Line 41-42: This sentence is hard to understand. If the authors would revise the previous sentence inline 40-41, then this sentence is redundant so that this sentence can be removed from the text.
15. Line 42-43: Would it be the same data or same database?
16. Statistical analysis: This section can be rewritten with sufficient detail. Some comments are the following:
17. Line 47-48: This sentence could be simplified. For example, we used the weekly percent of ILI.
18. Lines 48-49: I would suggest to write down the regression model and define the terms in the model.
19. Line 51: The model description and fitting method can be rewritten with model specification, as suggested in the previous comment.

20. Line 58: I would like to see the autocorrelation plot in the supplement.

21. Lines 59-60: I am afraid of the ”same steps” because I don’t find any steps before this line.

22. Line 60: Rate ratio - is it another model? What is the overdispersion problem?

23. Line 62-63: ”Analyses of the effects of swine flu pandemics were stratified ...” : I didn’t find any sentence about swine flu pandemics in the study population.

24. Lines 63-64: ”For the control analysis, all processes were replicated with cancer mortality as an outcome.” - does cancer mortality is weekly/monthly percent mortality during the same period as weekly/monthly percent suicide mortality?

25. Line 69: I would rewrite the study period in this section.

26. Line 72: ”There were monthly or seasonal variations in suicide, with the peak in the number of suicides...”- I would rewrite this sentence. For example, there were monthly **and** seasonal variations in suicide, with a peak in the rate of suicide **rate.**

27. Line 75-76: What are the statistical methods used to obtain these results?

28. Lines 77-89: I would summarize these results in a table.

29. Line 78: Why authors again considered ILI per 100 people?

30. Line 93: I would rewrite this sentence as ”In our study....”.

31. Line 94: I would be consistent in using novel influenza or A(H1N1)pdm09.

32. Line 96: Why did authors use influenza symptoms? Isn’t influenza infections?

33. Lines97-98: What do we mean by lost significance? I would write the sentence as ” there is not enough evidence to show a significant association between weekly suicidal rate and percent ILI after nine weeks from the pandemic.

34. Lines 98-99: What do we mean by the current trend? Moreover, I don’t see the authors discuss chronic diseases before this line.

35. Line 100: I would suggest keeping the results in the supplement.

36. Lines 102-103: This study discusses too many results/conclusions, so it would be better to specify what results from the previous research support the finding in this study.

37. Lines 103-106: I would recommend comparing the results of the same periodic data or mention the frequency of time series.

38. Line 108: ”However, a significant association was observed between depression and Influenza A infection.” - I would recommend rewriting this sentence with an appropriate transition word than ”However.”

39. Line 121: I would specify what are the significant results. Would it be a significant association or significant periodic association? What variables involved in these associations?

40. Line 134 - 135: I would write this sentence in the introduction.

41. Line 136: It is confusing to me that A(H1N1) has been considered as a new virus subtype in lines 134-135, but it is named as classic (previous) seasonal influenza A(H1N1). Why do both sentences contradicting?

42. ”This” - It is confusing to me what does this refer to?

43. I thought the authors selected weekly ILI cases from K-CDC for the complete analysis and mentioned in the results that after 2009 and until eight weeks, there was a significant association between suicide rate and percent ILI. If the HIN1 pandemic was in 2009, why did the authors couldn’t consider this data for their fact-checking?

44. Line 152: I would choose one word from ILI or influenza infections or influenza symptoms. As I understood, ILI is the most appropriate among these three words.

45. Line 155: This sentence arose a question of whether the current study considered suicide attempts or suicide mortality.

46. Line 159: ”However, this study featured a primarily ecological design,...” - Is it an observational study?

47. Lines 167-169: Would this ”original data” refer to the K-CDC database?

48. Line 170: This sentence could be written in the study population section.

49. Line 174: Could we drop ”large-sized”?

50. Lines 180-184: I would rewrite this sentence because this sentence has too many negative words such as, nevertheless, not definitely confirmed.

51. Lines 184-186: I would suggest check grammar.

52. Line 200: I would be consistent in using ILI instead of influenza symptoms.

53. Line 201: What would be the individual data?

54. Figures

    - Figures are blurred.

- I would write the caption for each figure below the figure.
- Figure 3: What is the y-axis? Is it a rate ratio of suicide? What is the grey line? Why is the label " % increment of influenza-like illness?" here?
- Figure 1: Why Figure 3 appears before presenting Figure 1? For the caption of figure 1, I would recommend to write the model and define the terms in the model. What is P? What is the time series adjusted?
- Figure 2: Does each boxplot include weekly suicide in each month? If this is the case, I will write this in the caption.
