# Supplementary material for: Fluctuations in influenza-like illness epidemics and suicide mortality: A time-series regression of 13-year mortality data in South Korea
Source: PLoS One. 2021 Feb 12;16(2):e0244596. doi: 10.1371/journal.pone.0244596 (PMC7880447; doi:10.1371/journal.pone.0244596)
Supplement: Attachment — Submitted filename: response to reviewer 20200701.docx [file pone.0244596.s003.docx]

**Author’s Response to the editors and reviewers’ comments:**

**Fluctuations in influenza-like illness epidemics and suicide mortality: A time-series regression of 13-year mortality data in South Korea**

We would like to thank the Editor and reviewers for their valuable feedback on our submission to the *PLos One*. We found your comments to be very helpful in improving the quality of our manuscript. Below are our responses to your comments.

-**Reviewer 1**

Reviewer #1:

• I strongly recommend using consistent words for suicide mortality and ILI rate. Throughout the manuscript, I read suicide attempts, influenza symptoms, influenza infections were used instead of suicide mortality, and ILI rate that could distract any reader. Another confusing word was novel A(H1N1)pdm09. Do we have a difference between novel A(H1N1)pdm09, classic novel A(H1N1)pdm09, the new novel A(H1N1)pdm09, (H1N1)pdm09? I think all are the same, but I would choose one name for the entire text. I strongly recommend using consistent words for suicide mortality and ILI rate.

: We unified all of the terms to “novel influenza A(H1N1)pdm09.”

• I was able to access ILI data, but I couldn't find the suicide rate data.

: The suicide data can be download from the mdis site: https://mdis.kostat.go.kr/eng.
All of the data can be downloaded after login. For access to my data, please refer to my Google Drive below.
https://drive.google.com/file/d/1wibMKITQVGdS1XACs0k0AnExEVqIT_g3/view?usp=sharing

1. Abstract:

Objective: The title is entirely different from the objective of the study. 1) As I understood, this study is only in Korea, so I would recommend reflecting this in the title. 2) title says influenza epidemics and suicide mortality, but the objective is about influenza infections and neuropsychiatric symptoms.

: To address your comment, we modified our title as follows:

*“Fluctuations in influenza-like ilness illness epidemics and suicide mortality: A time-series regression of 13-year mortality data in South Korea “*We also changed the aim as below:

*(page 4, lines 54-55)*

*“Aims: We explored the association between influenza epidemic and suicide mortality rates in a large population using a time-series regression of 13-year mortality data in South Korea. “*

Methods: I couldn’t understand the sentence that the regression coeffcient for suicide based on the percentage change of influenza.

: We analyzed the regression between number of suicide case and number of influenzas per 100 outpatient clinic visits. So, the regression coefficient was the mean(average) of ($\frac{\Delta suicide}{\Delta influenzas}$), and the change of suicide ($\Delta suicide$) was $\beta\times\Delta influenzas$ (percent change of influenzas). In general, ILI is the number of influenzas per 1,000 outpatient clinic visits, but we multiplied 10 to ILI to represent ILI per 100 outpatient visits (time series regression coefficient is generally reported as percent change of X).

2. Lines 2-9: This study focused on the population of Korea, so why wouldn’t the authors mention any previous study in Korea in the introduction?

: To address your comment, we added the following sentence:

*(page 5, lines 9-13)*

*“Especially in South Korea, one study dealt with the adverse psychiatric events after the treatment of H1N1, oseltamivir,[4] but evidence lacked for the association between infectious epidemic itself and neuropsychiatric symptoms. In addition, a new virus subtype labeled novel influenza A(H1N1)pdm09 first emerged in 2009, even though the influenza subtype of H1N1 existed before 2009. “*

3. Line 10: “In population studies …” I would suggest being specific (name it) about the population.

: We added specific details about the study population, as shown below.

*(page 5, lines 17-18)*

*“In a study conducted in South Korean South Korean children in hospital children with lower respiratory infections who were , aged less than 18 years, two distinct peaks in viral upper respiratory tract infection was noted yearly, each during April-May and October-November.[5]”*

4. Line 11: Do authors consider weekly/monthly suicide mortality? I would recommend saying the month instead of the spring season because seasons are different across countries.

: To improve clarity, we added the following:

*(page 5, lines 14-15)*

*“In population studies, the total numbers of suicide mortality exhibit have shown seasonal peaks, especially during the early spring, which usually falls inis late February to March in Korea.“*

5. Line 11: I would either use overlap or follow, not both.

: We deleted the term “follow” from our text.

6. Line 11-12: What are the traditional upper respiratory infections?

: We revised the sentence as follows:

*(Page 5, lines 17-21)*

*“These peaks overlap with seasonal peaks in epidemics of upper respiratory tract infection, which may affect thes nose, throat, sinus, and larynx. In a study conducted in South Korean South Korean children in hospital children with lower respiratory infections who were , aged less than 18 years, two distinct peaks in viral upper respiratory tract infection was noted yearly, each during April-May and October-November.[5]”*

7. line 14: I am not sure about using the “etiology of suicide.” Could we use the cause of suicide mortality? Moreover, if the prevention strategies for Korea, then authors could revise the sentence to reflect this in the sentence.

: We modified the sentence as below.

(Page5, lines 21-23)

*“The development of prevention strategies relies on an understanding of the cause of suicide mortality at a population level. “*

8. Line 16: new 16 influenza (H1N1) virus pandemic and A(H1N1)pdm09 are different?

: We unified all of the terms to “novel influenza A(H1N1)pdm09.”

9. Line 16: ”large” is a confusing adjective for the population.

: We changed “large” to “substantial number of.”

10. Line 23: I would say subject information is blinded.

: We stated that subject information was blinded prior to analysis.

11. Line 24: What does ”current study” refer to? Would it be a study in this paper? Correct me if I am wrong: I assume that this paper uses a retrospective study data.

: Current study means the study we’ve conducted. For clarity, the words related to study design were changed throughout manuscript, as appropriate.

12. Line 29-30: I would add ILI per 1000 visits weekly/monthly.

: We added the term “weekly” as you suggested.

Line 37: It is confusing that the introduction reads on neuropsychiatric symptoms, but this line picks only suicide. I would rewrite clearly whether this study considers only suicide mortality or the number of suicides per week or whatever appropriate throughout the text.

: Our analysis only included suicide and influenza. We did not analyze any other “neuropsychiatric symptoms.” The term “neuropsychiatric symptoms” in line 9 refers to a summary of a previous systematic review study. We wrote that sentence to generate our hypothesis.

13. Line 40-41: Correct me if I am wrong. I assume that the authors didn’t define the codes so that I would rewrite this sentence. For example, we chose cases with X60-X84 and Y870 related to intentional self-harm and sequelae of intentional self-harm, respectively.

: We admit that we did not define the codes. We modified that sentence to address your comment.

14. Line 41-42: This sentence is hard to understand. If the authors would revise the previous sentence inline 40-41, then this sentence is redundant so that this sentence can be removed from the text.

: As suggested, we revised the previous sentence in lines 40-41, and removed the mentioned sentence.

15. Line 42-43: Would it be the same data or same database?

: It came from the same database.

16. Statistical analysis: This section can be rewritten with sufficient detail. Some comments are the following:

17. Line 47-48: This sentence could be simplified. For example, we used the weekly percent of ILI.

: We revised the sentence according to your suggestion.

18. Lines 48-49: I would suggest to write down the regression model and define the terms in the model.

19. Line 51: The model description and fitting method can be rewritten with model specification, as suggested in the previous comment.

: Thank you for constructive comments. We wrote the formula for time series analysis with generalized additive model, as shown below.

$g \left( {suicide}_{t} \right)= \beta_{0}+ f_{1}\left( {ILI}_{t-l} \right)+ f_{2}\left( {Season}_{t} \right) +f_{3}\left( {Week}_{t} \right)$

where: $f_{s}(x_{t}$) = $\sum_{i=1}^{k} b_{i}(x_{t})\beta_{i}$ , $\beta_{i}$is the weight value applied to the $i$th basis function ($b_{i})$, so the $f_{s}(x_{t}$) is smooth function of covariates $x_{t}$. Suicide(*t*) is weekly(*t*) number of suicides, and ILI(*t-l*) is corresponding weekly ILI with lag time 0 to 8 weeks. Season and long-term trend by week are confounding factors.

20. Line 58: I would like to see the autocorrelation plot in the supplement.

: We added partial ACF, as shown below. We also attached the basic decomposition plot.

21. Lines 59-60: I am afraid of the ”same steps” because I don’t find any steps before this line.

: We simulated the degree of free dome (df) of GAM spline parameter for minimal AIC value (from line 52 to 60), and used the same simulation to find the optimal lag week. As you suggested, we removed the term the “step” and added “simulation.”

22. Line 60: Rate ratio - is it another model? What is the overdispersion problem?

: Rate ratio is another name for ($\frac{\Delta suicide}{\Delta influenzas}$), and rate ratio is the ratio between suicide rate and ILI rate. Overdispersion means the assumptions of the model were not met due to wide range of variations, so we cannot trust its output within statistical assumption. Therefore, we used quasi-Poisson model to deal with it (reference :Overdispersion, and how to deal with it in R and JAGS (requires R-packages AER, coda, lme4, R2jags, DHARMa/devtools) Carsten F. Dormann 07 December, 2016)

23. Line 62-63: ”Analyses of the effects of swine flu pandemics were stratified …” : I didn’t find any sentence about swine ƒu pandemics in the study population.

: Swine flu pandemics were stratified by the year of epidemics, as shown below.

Response to Reviewer figure 3 The number of influenza virus isolates by subtype in South Korea, 2000–2017

Note: The numbers of influenza virus cases were sourced from the report of infectious diseases surveillance yearbook reported form the Korea Centers for Disease Control and Prevention (K-CDC) in 2017 [1]. A(H1N1)pdm09, marked in orange color, emerged since 2009.

24. Lines 63-64: ”For the control analysis, all processes were replicated with cancer mortality as an outcome.” - does cancer mortality is weekly/monthly percent mortality during the same period as weekly/monthly percent suicide mortality?

: Yes we used the same method for cancer mortality (weekly).

25. Line 69: I would rewrite the study period in this section.

: We rewrote the study period as “Data from August 2004 to December 2017 were used in our analysis.”

26. Line 72: ” There were monthly or seasonal variations in suicide, with the peak in the number of suicides…”- I would rewrite this sentence. For example, there were monthly and seasonal variations in suicide, with a peak in the rate of suicide rate.

: We changed the term “number” to “rate” according to your comment.

27. Line 75-76: What are the statistical methods used to obtain these results?

: Linear regressions were conducted each month, and we added the phrase “by linear regression.”

28. Lines 77-89: I would summarize these results in a table.

: We have summarized the results in a table, as shown below. But we would like summarize these results as a figure instead. We ask for your kind understanding of our purpose, as we would like to show the trend of lag effect in a figure.

| lags | RR (95% CI) |
| --- | --- |
| lag 0 | 1.007 (1.003–1.012) |
| lag 1 | 1.007 (1.003–1.011) |
| lag 2 | 1.006 (1.003–1.010) |
| lag 3 | 1.006 (1.002–1.009) |
| lag 4 | 1.005 (1.001–1.008) |
| lag 5 | 1.004 (1.001–1.007) |
| lag 6 | 1.004 (1.001–1.007) |
| lag 7 | 1.004 (1.001–1.007) |
| lag 8 | 1.003 (1.000–1.007) |
| lag 9 | 1.003 (0.999-1.006) |

29. Line 78: Why authors again considered ILI per 100 people?

: We made a mistake there. To clarify, we just used ILI in the sentence.

30. Line 93: I would rewrite this sentence as ”In our study….”.

: We modified it as follows:

*“In our study using population data, we identified a significant positive association between ILI and suicide beginning in 2009, the year in which a novel influenza A(H1N1)pdm09 virus provoked a worldwide pandemic.”*

31. Line 94: I would be consistent in using novel influenza or A(H1N1)pdm09.

: We unified the term to “A(H1N1)pdm09” throughout the manuscript.

32. Line 96: Why did authors use influenza symptoms? Isn’t influenza infections?

: We changed the term to “influenza infections.”

33. Lines97-98: What do we mean by lost significance? I would write the sentence as “there is not enough evidence to show a significant association between weekly suicidal rate and percent ILI after nine weeks from the pandemic.”

: We changed the corresponding sentence according to your suggestion.

34. Lines 98-99: What do we mean by the current trend? Moreover, I don’t see the authors discuss chronic diseases before this line.

: We deleted the sentence.

35. Line 100: I would suggest keeping the results in the supplement.

: We presented the results as below. However, our study design did not focus on cancer mortality, and cancers have various disease sub-types. Therefore, we did not show this figure in the supplement. We ask for your kind understanding of our purpose.

36. Lines 102-103: This study discusses too many results/conclusions, so it would be better to specify what results from the previous research support the finding in this study.

: For improved clarity, we modified the sentence as follows:

*“Corresponding to our results which indicated a significant positive association between ILI number and suicide mortality, one large prospective cohort study[8] conducted using the Danish Civil Registration System showed the similar results, supporting our study findings.”.”*

37. Lines 103-106: I would recommend comparing the results of the same periodic data or mention the frequency of time series.

: The analysis mentioned in the text was conducted based on the data of Danish Civil Registration System from 1980 to 2011. And, unlike our analysis, it utilized the individual follow-up data, which made it difficult to make a direct comparison with the frame of our analysis. However, for clarity, we modified the sentence as follows:

*(page 9, lines 111-114)*

*“Among 7,221,578 Danish individuals from 1980 to 2011, hospitalization by viral infection increased the suicide mortality incidence by 1.26 times, compared with control populationa lack of any infection history, after adjusting for sex, age, calendar period, cohabitation status, socioeconomic status, and comorbidity.”*

38. Line 108: “However, a significant association was observed between depression and Influenza A infection.” - I would recommend rewriting this sentence with an appropriate transition word than ”However.”

: We modified the sentence as follows:

(page 9, lines 114-118)

*“Another prior study[9] of various anti-viral antibody titers in 257 individuals from the U.S. S identified a significant relationship between suicidal behavior and influenza B, but not influenza A infection;, and a significant association was observed between depression and Influenza A infection.“*

39. Line 121: I would specify what are the significant results. Would it be a significant association or significant periodic association? What variables involved in these associations?

: We clarified this part by using the term “association.”

40. Line 134 - 135: I would write this sentence in the introduction.

: We added the following sentence to the introduction section.

*(page 5, lines 11-13)*

*“In addition, a new virus subtype labeled novel influenza A(H1N1)pdm09 first emerged in 2009, even though the influenza subtype of H1N1 existed before 2009”.”*

41. Line 136: It is confusing to me that A(H1N1) has been considered as a new virus subtype in lines 134-135, but it is named as classic (previous) seasonal influenza A(H1N1). Why do both sentences contradicting?

: During the overall revision of the manuscript, corresponding contents have been removed and re-written as follows:

(page 10-11, lines 150-153)

*“The biological characteristics of a new virus subtype labeled novel influenza A(H1N1)pdm09 were considerably different from those of the classic (previous) seasonal influenza A(H1N1), which exhibited higher severity in clinical symptoms and strong transmission tendency [14, 17]. “*

42. ”This” - It is confusing to me what does this refer to?

: We removed the term “this” from our manuscript.

43. I thought the authors selected weekly ILI cases from K-CDC for the complete analysis and mentioned in the results that after 2009 and until eight weeks, there was a significant association between suicide rate and percent ILI. If the HIN1 pandemic was in 2009, why did the authors couldn’t consider this data for their fact-checking?

: We agree with your comment. We also want to fact-check using pathologically confirmed cases. However, the Korean government did not report on the number of ILI cases based on pathologic information.

44. Line 152: I would choose one word from ILI or influenza infections or influenza symptoms. As I understood, ILI is the most appropriate among these three words.

: We corrected the terms related to overall use of influenza to “ILI” throughout our manuscript.

45. Line 155: This sentence arose a question of whether the current study considered suicide attempts or suicide mortality.

: We corrected our manuscript to indicate “suicide mortality.”

46. Line 159: ”However, this study featured a primarily ecological design,…” - Is it an observational study?

: To reduce confusion, we changed the sentence as follows:

*(page 13, lines 215-217)*

*“However, this study utilized the grouped data, and individual causality could not be interpreted. During modeling, we could not consider important confounders and mediators, including socio-economic and life-style factorss or comorbidities.”*

47. Lines 167-169: Would this ”original data” refer to the K-CDC database?

: Yes. To reduce confusion, modified the sentence as follows:

(page13, lines 223-225)

“However, we could not perform a gender- specific analysis, since there was no gender- specific information on ILI rate that could be obtained from the original K-CDC data.”

48. Line 170: This sentence could be written in the study population section.

: We also added the contents to the study population section as follows:

(page 6, lines 33-37)

*“As the information regarding virus genotyping was unavailable, we could not distinguish between H1N1 infection and other flu-like illness, however, we obtained information about ILI outpatient visits (per 1,000 people) weekly ranging from the 35th International Organization for Standardization (ISO) week of in 2004 to the 52th ISO week of in 2017.”*

49. Line 174: Could we drop ”large-sized”?

:We deleted the term “large-sized” from the manuscript.

50. Lines 180-184: I would rewrite this sentence because this sentence has too many negative words such as, nevertheless, not definitely confirmed.

: We modified the sentence as follows:

(page 14, lines237-241)

*“The U.S. Center for Disease Control and Prevention[34] and the Institute of Medicine Committee to Review Adverse Effects of Vaccines[35] stated that there is no confirmed adverse effect of influenza vaccine on any of neuropsychiatric adverse events, including brain inflammation, encephalopathy, Acute Disseminated Encephalomyelitis, optic neuritis[36], and Guillain-Barre Syndrome[37].”*

51. Lines 184-186: I would suggest check grammar.

: We corrected it.

52. Line 200: I would be consistent in using ILI instead of influenza symptoms.

: We corrected the overall use of influenza-related terms to “ILI” throughout our manuscript.

53. Line 201: What would be the individual data?

: To improve clarity, we changed the term to “individual-leveled data.”

54. Figures

• Figures are blurred.

: We attached more high resolution images (300 dpi) for the figures.

• I would write the caption for each figure below the figure.

: Yes, we added the captions for each figure in the figures legends

• Figure 3: What is the y-axis? Is it a rate ratio of suicide? What is the grey line? Why is the label “ % increment of influenza-like illness?” here?

: Y-axis is the rate ratio of suicide. Gray area means 95% confidence interval, and gray line means vertical and horizontal subline. For x-axis, gray line indicates lag (weeks).

• Figure 1: Why Figure 3 appears before presenting Figure 1? For the caption of figure1, I would recommend to write the model and define the terms in the model. What is P? What is the time series adjusted?

: It was presented in that order as Figure 1 appears in line of 83, Figure 2 appears in line of 84, and Figure 3 appears in line of 98. We wrote down the formula of time series analysis with generalized additive model (Figure 1), as shown below.

“$g \left( {suicide}_{t} \right)= \beta_{0}+ f_{1}\left( {ILI}_{t-l} \right)+ f_{2}\left( {Season}_{t} \right) +f_{3}\left( {Week}_{t} \right)$

where: $f_{s}(x_{t}$) = $\sum_{i=1}^{k} b_{i}(x_{t})\beta_{i}$ , $\beta_{i}$is the weight value applied to the $i$th basis function ($b_{i})$, so the $f_{s}(x_{t}$) is smooth function of covariates $x_{t}$. Suicide(*t*) is the weekly(*t*) number of suicides, and ILI(*t-l*) is the corresponding weekly ILI with lag time 0 to 8 weeks. Season and long-term trend by week are confounding factors.” We would like insert this sentence in the methods section.

• Figure 2: Does each boxplot include weekly suicide in each month? If this is the case, I will write this in the caption.
: We added the following: “Figure 2. Monthly variation in weekly numbers of suicides during study period.”

Reviewer #2: The authors investigated explored the association between influenza infection and suicide rates in a large population using a 57 time-series regression of 13-year mortality data This is an exceptionally timely and commendable work. Several short-comings are well acknowledged in the limitations section. The authors are urged to address the following points

1. Discuss implications and not only list differences between studies that find longer lags and, as this study, that found shorter lags (here longer than 9 weeks results are not significant). The implications begin with biology- as longer lags imply a sequence of events of cumulation of risk before the maximum predictive association becomes manifest. The authors should discuss the potential of priming of immune cells , including resident immune cells in the brain- process that takes time, and makes the individual more vulnerable to innocuous immune, mechanical and psychological triggers. They could also discuss at a macro level differences between the first Danish study and their studies

: Thank you for raising an excellent point. We added the following contents to address your comment.

*(pages 11-12. Lines 173-183)*

*“In our study, we found lags shorter than 8 weeks with significantly positive association between ILI and suicidal behavior, and the result from Denmark study[8] did not applied apply any lag, which could imply that be interpreted that their result represent more immediate effect of infection and suicide death. Neuroinflammation can be one of the explanations linking the virus infection and suicidal behavior. Immediate neuroinflammatory changes, including sudden increase in pro-inflammatory cytokines, can exhibit brief symptoms;, whereas neuroinflammatory priming, both peripheral and central signaling cells - particularly microglia, exhibit a long-term and derivative change. After stress, primed microloglia produce new “silent” immune machinery, which causes immune insult and a hyperinflammatory response.[26] Beyond biological mechanisms, the lag between ILI epidemics and suicide include population level analytics, such as window time among exposure and clinical symptoms, or hospitalization”.*

2. Avoid the term suicidality. Although there is some terminological confusion, and examples of the use of the term the way the authors have used it so far, for majority of suicide researchers suicidality refers to suicidal ideation not behavior (behavior includes suicide = death by suicide, and non-fatal suicide attempts). The authors would do well to refer to previous studies on attempts as "suicidal behavior" and not suicidality (e.g. citation by Arling), and on studies on death by suicide- as suicide

: We corrected the terminology throughout the entire manuscript.

3. In arguing that the results are not the effect of antivirals the authors write that a Longitudinal study did not find associations with antivirals, but did find associations with antibiotics. It is immortal to report, if data are available - what that percentage of viral infections lead to bacterial complications treated with antibiotics , and what of the percentage of confirmed influenza individuals end up on antibiotics in Korea. If antibiotics may increase risk of suicide - e.g. via reducing the immunomodulatory funciton of the gut microbiota, then it is possible, if large numbers of individuals end up being treated for (real or presumed) respiratory bacterial complications of Influenza, that the antibacterial treatment contributes to results.

: It has been known that the total monthly rate of antibiotic use was highly cross-correlated with the monthly detection rate of influenza virus in Korea (cross-correlation coefficient 0.47). Therefore, it is possible that people who had viral infections might have undergone antibiotic treatment. However, there is a lack of evidence that antibiotics increase the risk of suicidal behavior. Among the antibiotics, fluroquinolone was reported to have side effects on mental health by the U.S. FDA; however, suicidal behavior was not included as a side effect. We also had no information regarding antibiotic use in our population.

We added the following details to our discussion section.

(page 10, lines 142-148)

*“It has been known that total monthly rate of antibiotic use was highly cross-correlated with the monthly detection rate of influenza virus in South Korea (cross-correlation coefficient 0.47),[16], so it is possible that people who had viral infection might have antibiotic treatment. However, there is a lack of evidence that antibiotics increase the risk of suicidal behavior. Among the antibiotics, fluroquinolone was informed toreported to have some side effects on mental health by according to the U.S. Food and Drug Administration; , however, suicidal behavior was not included as the a side effect”*

4. There is a bit of lack of precision on citations being used, so that when referring the kynurenines- the authors cite nonspecific articles rather that very specific ones

a): Sublette ME, Galfalvy HC, Fuchs D, Lapidus M, Grunebaum MF, Oquendo MA, Mann JJ, Postolache TT. Plasma kynurenine levels are elevated in suicide attempters with major depressive disorder. Brain Behav Immun. 2011 Aug;25(6):1272-8. doi:10.1016/j.bbi.2011.05.002. Epub 2011 May 14. PMID: 21605657; PMCID: PMC3468945.

b: Brundin L, Sellgren CM, Lim CK, Grit J, Pålsson E, Landén M, Samuelsson M, Lundgren K, Brundin P, Fuchs D, Postolache TT, Traskman-Bendz L, Guillemin GJ, Erhardt S. An enzyme in the kynurenine pathway that governs vulnerability to suicidal behavior by regulating excitotoxicity and neuroinflammation. Transl Psychiatry. 2016 Aug 2;6(8):e865. doi: 10.1038/tp.2016.133. PMID: 27483383; PMCID: PMC5022080.

c: Okusaga O, Duncan E, Langenberg P, Brundin L, Fuchs D, Groer MW, Giegling I, Stearns-Yoder KA, Hartmann AM, Konte B, Friedl M, Brenner LA, Lowry CA, Rujescu D, Postolache TT. Combined Toxoplasma gondii seropositivity and high blood kynurenine--Linked with nonfatal suicidal self-directed violence in patients with schizophrenia. J Psychiatr Res. 2016 Jan;72:74-81. doi: 10.1016/j.jpsychires.2015.10.002. Epub 2015 Oct 9. PMID: 26594873.

: We modified all of the citations and specified their locations according to your suggestion.

For the immune activation and suicidal behavior- the citations that the author chose are neither the first, nor the most meta-analysis. We suggest replacing them either with the historically first - in the blood, in the CSF, in the brain or with the most recent several meta-analyses.

: We found the most recent and comprehensive meta-analysis regarding inflammation and major psychiatric disorder, and added it as a reference (Yuan, N., Chen, Y., Xia, Y. et al. Inflammation-related biomarkers in major psychiatric disorders: a cross-disorder assessment of reproducibility and specificity in 43 meta-analyses. Transl Psychiatry 9, 233 (2019). <https://doi.org/10.1038/s41398-019-0570-y>).

5. The authors correctly acknowledge overlaps between influenza and other immune and non immune mediated conditions occurring in spring such as pollen exposure and light- but it would be important to cite articles for those, and discuss lags involved in this study relative to the articles on lags of light exposure. The authors may also make the connection between influenza, sinus infections and allergic rhinitis by mentioning the nose/ upper airway to brain pathways for viruses, immune signals and immune cells, further affecting brain structure and function.

: As you suggested, we added the following details:

(page 11, lines 165-172)

*“Also, a respiratory virus affecting the nose and upper respiratory tract, including sinus infections and allergic rhinitis, has been mentioned as being responsible for central nervous system(CNS) pathologies; , viruses such as influenza, coronavirus, and human respiratory syncytial virus are known as a neuropathological virus. Through the neurons which innervate the peripheral organ and olfactory neurons, these viruses enter the CNS, reach the whole brain and cerebrospinal fluid (CSF), and cause demyelination. Simultaneously, primary glial cultures under these conditions have shown the secretion of cytokines in CSF , such as IL-6, IL-15, TNF-α, and CXCL9/10.[25].” “However, our result indicating seasonal variations in suicidal deaths should be interpreted with other seasonal factors, such as sunlight and pollen exposure. The results from about 43,000 people in Finland showed a significant peak in of suicide deaths between May to and June, which had tend to have the longest sunlight exposure of the year, and the lag period for this association was around 1 month.[27] Contrary to this study, our results showed significant peak of suicidal mortality from March to May, which shows moderate sunlight exposure. Usually, the solstice in South Korea is onKorea happens in December, so even; therefore, even if though we consider the maximum lag of to be 8 weeks, there is a slight chancestill a possibility for of period overlap due to the sunlight exposure. In addition, pollen counts were suspected to be the a factor associated with suicide rate,[28]; however, a study with data from 42 counties of the continental U.S. did not replicated the significant association.[29]. Furthermore, our current model controlled seasonal effects using GAM and time series model, and those adjustments did not attenuate the association between ILI fluctuation and suicide.”* 6. The differences between before 2009 pandemic and post 2009 pandemic require additional considerations- were there socioeconomic implications or individual psychological stress related to the pandemic contributing to risk. Even if speculative, this component will be very important for COVID-19. In that regard, at this stage the authors would be advised to comment on how the influenza pandemic was different from current COVID 19, and what expectations this article on influenza generates for pandemic of respiratory neurotropic viruses in general

: We added the following contents to address your comment.

(page 12-13, lines 198-207)

*“The socio-economic difference of between times before the 2009 pandemic and post-2009 pandemic would should also be considered. In our previous study using national statistics in South Korea, we found an increased suicide rate during the 2008 global financial crisis in 2008. We could observe a rise in suicidal mortality, especially among elderly male office workersrs and managers.[30] Possible interaction may exist between job status, sex, and ILI on with suicidal deaths in our data. The novel coronavirus (COVID-19) outbreak in 2019 (COVID-19) would differ from novel Influenza A(H1N1)pdm09, in terms of regarding thethe proportion of affected the people affected, itheirt’s social influence, and the absence of the vaccine and treatment, which may trigger suicidal behavior in more socio-economic context. Further analyses for the adverse mental health outcomes, including suicidal behavior from thesedue to these kindstypes of respiratory neurotropic viruses, are warranted.”*

1.	KCDC. KCDC Releases 2017 Infectious Disease Surveillance Yearbook: Korea Centers for Disease Control and Prevention; 2017 [cited 2019 26th April]. Available from: <https://www.cdc.go.kr/CDC/eng/info/CdcKPress.jsp?menuIds=HOME002-MNU0576-MNU0586&fid=8652&q_type=&q_value=&cid=123003&pageNum=1>.
