# Supplementary material for: Fluctuations in influenza-like illness epidemics and suicide mortality: A time-series regression of 13-year mortality data in South Korea
Source: PLoS One. 2021 Feb 12;16(2):e0244596. doi: 10.1371/journal.pone.0244596 (PMC7880447; doi:10.1371/journal.pone.0244596)
Supplement: Attachment — Submitted filename: response to reviewer 20201128.docx [file pone.0244596.s005.docx]

**Author’s Response to the editors and reviewers’ comments:**

**Fluctuations in influenza-like illness epidemics and suicide mortality: A time-series regression of 13-year mortality data in South Korea**

We would like to thank the Editor and reviewers for their valuable feedback on our submission to the *PLos One*. We found your comments to be very helpful in improving the quality of our manuscript. Below are our responses to your comments.

-**Reviewer 1**

Reviewer #1:

(General comment) thank you for incorporating my comments to revise the manuscript. I found some revisions are missing and highlighted in red color. I am still worried that the figures are blurred.

(Response) Thank you for your consideration. I responded to comments as highlighted in ‘red color’. And update our figures’ resolution (more than 300 dpi).

Comments 1) Statistical analysis: This section can be rewritten with sufficient detail. Some comments are the following:

1) The generalized additive model is missing the error term. I’m also missing the method of estimation.

(response) Thank you for your detail comment. I add error term of time as below.

$$g \left( {suicide}_{t} \right)= \beta_{0}+ f_{1}\left( {ILI}_{t-l} \right)+ f_{2}\left( {Season}_{t} \right) +f_{3}\left( {Week}_{t} \right) + \varepsilon(t)$$

2) Could you please comment/interpret the partial ACF?

(response) I writted sentence as “Then, after controlled seasonality, The partial autocorrelation plot of residuals showed no cyclic pattern.” in method section (line 68).

3) It seems that choosing the degrees of freedom for the smoothing splines is not clearly written. Have you chosen the degrees of freedom based on AIC?

(response) Yes I did. I used AIC to choose optimal df for smoothing splines. “Every step of model building were simulated by degree of freedom (df) from 2 to 200 in the generalized linear model or GAM, and graphical approach was used to detect the optimal df of GAM spline parameter for minimal AIC” in line 64.

Comments 2) I would write ’using linear regression’.

(response) Yes, we used linear regression, but other reviewer asked us to clarify the ‘over-dispersion’ problem, so we added words of “quasi-Poisson regression”. Please kindly understand our purpose to that.

Comments 3) I agree that the figure is much better than the table, but the figure is blurred

I added some additional note to figure 1:

“Figure 1. Weekly numbers of suicides and influenza-like illnesses per 100 outpatients from 2004 to 2017.”

“Note: β (Beta) indicates the change in weekly suicide numbers, considering the % increment in influenza-like illness cases; red dot line show weekly number of suicide after adjusting time series factors, and red shadow indicates 95% confidence interval of the estimated weekly suicide rate, which was assessed using a generalized additive model; ,blue line show influenza like illness per 100 outpatients without lag time, and horizontal line indicates the year 2009.”

Comments 4) . Lines 102-103: This study discusses too many results/conclusions, so it would be better to specify what results from the previous research support the finding in this study. I thought that the significant positive association between ILI number and suicide mortality is only after the 2009 pandemic.

(response) we edited sentence as your comment

“In our study using population data, we identified a significant positive association between ILI and suicide mortality only after the 2009 pandemic, the year in which a novel influenza A(H1N1)pdm09 virus provoked a worldwide pandemic.”

Comments 5) Line 108: ”However, a significant association was observed between depression and Infuenza A infection.” - I would recommend rewriting this sentence with an appropriate

transition word than ”However.” the rewritten sentence seems too long

(Response) I edited that sentence to “but 9 weeks after did not.”

Comments 6) Figures are still blurred

(Response) I added note to figure 1.

Comments 7) Figure 1: Why Figure 3 appears before presenting Figure 1? For the caption of figure 1, I would recommend to write the model and define the terms in the model. What is P? What is the time series adjusted? the model is missing an error term error(t)

(response) I added error term of time.

$$g \left( {suicide}_{t} \right)= \beta_{0}+ f_{1}\left( {ILI}_{t-l} \right)+ f_{2}\left( {Season}_{t} \right) +f_{3}\left( {Week}_{t} \right) + \varepsilon(t)$$

Comments 8) I thought the authors changed number of suicide to suicide rate.

(response) I am sorry that the Y axis mean actual number of suicide, but the suicide rate may go similar because that suicide number is getting from all of Korean death certification records.
